# Ultra-Fast Evaluation of Protein Energies Directly from Sequence

**DOI:** 10.1371/journal.pcbi.0020063

**Published:** 2006-06-16

**Authors:** Gevorg Grigoryan, Fei Zhou, Steve R Lustig, Gerbrand Ceder, Dane Morgan, Amy E Keating

**Affiliations:** 1 Department of Biology, Massachusetts Institute of Technology, Cambridge, Massachusetts, United States of America; 2 Department of Physics, Massachusetts Institute of Technology, Cambridge, Massachusetts, United States of America; 3 DuPont Central Research and Development, Experimental Station, Wilmington, Delaware, United States of America; 4 Department of Material Science and Engineering, Massachusetts Institute of Technology, Cambridge, Massachusetts, United States of America; 5 Department of Material Science and Engineering, University of Wisconsin, Madison, Wisconsin, United States of America; Cornell University, United States of America

## Abstract

The structure, function, stability, and many other properties of a protein in a fixed environment are fully specified by its sequence, but in a manner that is difficult to discern. We present a general approach for rapidly mapping sequences directly to their energies on a pre-specified rigid backbone, an important sub-problem in computational protein design and in some methods for protein structure prediction. The cluster expansion (CE) method that we employ can, in principle, be extended to model any computable or measurable protein property directly as a function of sequence. Here we show how CE can be applied to the problem of computational protein design, and use it to derive excellent approximations of physical potentials. The approach provides several attractive advantages. First, following a one-time derivation of a CE expansion, the amount of time necessary to evaluate the energy of a sequence adopting a specified backbone conformation is reduced by a factor of 10^7^ compared to standard full-atom methods for the same task. Second, the agreement between two full-atom methods that we tested and their CE sequence-based expressions is very high (root mean square deviation 1.1–4.7 kcal/mol, *R^2^* = 0.7–1.0). Third, the functional form of the CE energy expression is such that individual terms of the expansion have clear physical interpretations. We derived expressions for the energies of three classic protein design targets—a coiled coil, a zinc finger, and a WW domain—as functions of sequence, and examined the most significant terms. Single-residue and residue-pair interactions are sufficient to accurately capture the energetics of the dimeric coiled coil, whereas higher-order contributions are important for the two more globular folds. For the task of designing novel zinc-finger sequences, a CE-derived energy function provides significantly better solutions than a standard design protocol, in comparable computation time. Given these advantages, CE is likely to find many uses in computational structural modeling.

## Synopsis

Many applications in computational structural biology involve evaluating the energy of a protein adopting a specific structure. A variety of functions are used for this purpose. Statistical potentials are fast to evaluate but do not have a clear biophysical basis, whereas physics-based functions consist of well-defined terms that can be costly to compute. This paper describes how the theory of cluster expansion, originally developed to describe the energies of alloys, can be applied to generate a physical potential for proteins that is extremely fast to evaluate. Cluster expansion is a way of representing a property of a system as a discrete function of its degrees of freedom. In this paper, it is used for the problem of protein design, where the energy is determined by the identities and conformations of amino acids at different sites on a fixed protein backbone. Application of cluster expansion to three small protein folds—the α-helical coiled coil, the zinc finger, and the WW domain—shows that protein sequence can be mapped directly to energy using a surprisingly simple function that maintains high accuracy. Promising results on these small systems suggest that the theory may have utility for macromolecular modeling more generally.

## Introduction

Protein structure prediction, homology modeling, fold recognition, and design, including the prediction and design of macromolecular interactions, are among the most complex and essential problems in contemporary computational structural biology. Proteins are critical players in the cell, and their function is dictated by their structure. Because the number of proteins with known sequence far exceeds the number with known structure, an ability to predict structure from sequence would be extremely valuable. On the other hand, designing proteins with specific structure and function is also important because of the usefulness of proteins as reagents and therapeutics [1].

At the heart of any computational approach to protein design or structure prediction lies the problem of determining the fitness (effective energy) of a particular protein in a given conformation or state. Depending on the method used, this effective energy may correspond to different physical quantities, e.g., stability, solubility, binding affinity, catalytic efficiency, or a combination thereof. In protein design, the goal is to optimize this fitness in the large space of possible amino-acid sequences. In the fold-recognition approach to structure prediction (also called threading), the goal is to identify the most suitable structure for a particular sequence, given a library of known folds. In both cases, the complexity of the problem imposes two sometimes conflicting requirements on the energy function used: physical accuracy and computational efficiency.

There are two major classes of fitness functions used in the fields of structure prediction and design. Lazaridis and Karplus [2] refer to these as statistical effective energy functions (SEEFs) and physical effective energy functions (PEEFs). SEEFs are derived from databases of proteins with known structures and describe the distribution of residues (or atoms) at different distances, solvent exposure, and sometimes more complicated measures, such as local atom density or relative orientation of secondary structure elements [3]. These terms are treated as effective potentials for calculating the energy of a protein in a given conformation. Most statistical energy functions include up to pair interactions [4–6]. However, it has been suggested that pairwise statistical energy functions may not be suitable for protein design or fold prediction [7,8], so some SEEFs include higher-order terms [8–10]. The advantages of SEEF methods lie in their computational efficiency, simplifying abstraction from details, and ability to implicitly capture effects such as desolvation, loss of entropy, and the hydrophobic effect, which are hard to account for explicitly. To gain these benefits, accuracy and physical interpretability are compromised.

PEEFs use atomic-level representations to capture underlying physical phenomena and approximate the free energy of the studied system. Some of the terms commonly included in PEEFs are van der Waals interactions, electrostatic interactions, hydrogen bond energies, dihedral angle torsion energies, atomic desolvation energies, and solvent-accessible surface area–dependent or volume-dependent estimates of the hydrophobic effect [2,11–13]. Some attempts have also been made to model side-chain entropy [14]. The advantage of PEEFs is that they have the potential to provide a more comprehensive understanding of the observed phenomena. The disadvantages are that much of the underlying physics is difficult to account for quantitatively, and when it is possible to do so, it is usually computationally expensive. An optimal energy function would have the simplicity and computational efficiency offered by SEEFs while retaining the theoretical rigor and physical interpretability of PEEFs.

A protein's behavior is a function of its sequence, given a defined environment. In particular, the energy required for a protein to fold to a given state or conformation (a quantity of central importance for protein design and structure prediction problems) is a function of its sequence regardless of the complexity of the underlying physics that determines that energy. In this paper we present a general method by which the energy of a protein on a fixed backbone, given by an arbitrary energy function, can be accurately expressed as a simple function of its sequence. In principle, this method can be applied in conjunction with any energy function, the only limitation on the complexity being that it must be possible to generate energies for enough training sequences with reasonable computational effort. We illustrate an application in which the calculated molecular mechanics energy of a protein, with a continuum treatment of solvation, can be mapped to a simple function of sequence that is extremely fast to evaluate and that maintains high accuracy. We find that the number of training sequences required to compute this mapping is significantly lower than would normally be adequate for sequence–space searches done in protein design. Furthermore, the resulting expansion retains, and in certain ways enhances, physical interpretability.

In the following sections, we first present an overview of the theory of cluster expansion (CE) and detail its application to protein structural modeling. We point out how the expansion consists of terms that are conceptually familiar to biochemists. We then go on to apply the method to three protein systems: the α-helical coiled coil, the zinc finger, and the WW domain. For each system, we show that CE can derive useful yet highly simplified energy expressions. We conclude with a direct demonstration of the power of CE in protein design.

### Theory

We seek to express the energy of a protein folding to a particular conformation as a function of its sequence. To accomplish this, we employ the technique of cluster expansion. CE is a method for representing a property (in this case, energy) that depends on discrete and topologically ordered degrees of freedom in a system [15]. The method finds its origin in alloy theory, in which very expensive ab initio calculations are required to accurately capture material properties, and only computations on a small number of atomic arrangements with relatively small unit cells are possible [15,16]. The CE is essentially a parameterization of the energy in terms of discrete variables that give the occupancy of each lattice point in the crystal. When the occupation variable is a spin variable (σ_i_ = +1 or −1), the CE takes on the form of a generalized Ising model. This approach has proven itself highly accurate in predicting alloy phase diagrams [17–19] and in identifying novel low-energy crystal structures [20,21].

In its more general form, CE is an expansion of the energy in a set of linearly independent basis functions that span the relevant configuration space (e.g., all possible distributions of atoms A and B on a crystal lattice, or all possible amino-acid sequences on a protein backbone). In most forms, the basis set of the CE is mathematically complete by construction, and a full expansion will result in a perfect representation of the energy. Truncated expansions may have practical utility, however. The use of a truncated CE to model the energy is analogous to using any truncated expansion in basis functions (e.g., plane waves or spherical harmonics) to represent a complex unknown function. The goal in developing an effective CE is to identify a truncated expansion that, when fit to a training set of data, provides an accurate mapping between degrees of freedom and energy using a minimal number of parameters.

**Figure 1:**
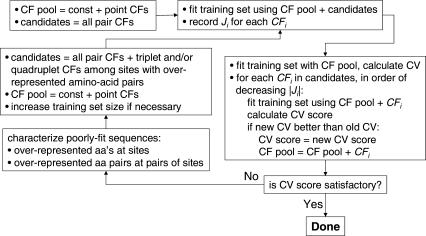
The Procedure for Fitting a Cluster Expansion Cluster functions (CFs) capture the contribution of a particular set of amino acids (aa) at a set of sites. Point, pair, and triplet CFs contain the contributions of amino acids at single sites, pairs of sites, triplets of sites, etc. The energetic contribution of any cluster function *CF_i_* is denoted by the variable *J_i_*. CV score designates the cross-validation root mean square error (i.e., the average error with which the energy of each sequence is predicted when left out of the fit), and its behavior serves as a measure of parameter significance. The goal of the fitting procedure is to find an optimal pool of CFs with which to expand the energy. Point and constant (const) CFs are always included and thus form an initial pool of CFs. In the next step, all pair CFs are considered as candidates. In order to asses the relative importance of candidate CFs, they are initially all added into the fit and their corresponding *J_i_*'s are stored. The candidates are then visited one by one in the order of decreasing |*J_i_*| and considered for inclusion into the CF pool. Candidates are included if they reduce the CV score. If the final CV score upon trying all pair CFs is not satisfactory, the list of candidates is appended with higher-order terms, and the procedure is repeated. Details are provided in Materials and Methods.

We have recently pioneered the use of CE for describing protein energetics [22]. To do so, we make a correspondence between an alloy lattice and a protein backbone, and between alloy constituent elements and amino acids. Whereas alloy problems are typically solved for two or three possible species per site, the complete collection of natural amino acids requires 20 species per site. Such a dramatic increase in phase space requires some reformulation of the CE implementation typically used for alloys. The general idea is to define a set of basis functions that correspond to the energetic contributions of single amino acids at single sites, pairs of amino acids at pairs of sites, triples of amino acids at sets of three sites, and so on. If intuition holds, the lower-order terms in this expansion will be more important than the higher-order ones, and a truncated expansion will be sufficient to represent the energy. In practice, given a set of training sequences and their energies, the CE is derived by starting with lower-order terms and successively considering higher-order contributions until a fit of the expansion to the data gives adequate performance when tested under cross validation. This process is outlined in the flowchart in Figure 1 and elaborated in the Materials and Methods. A formal description of the theory of CE as we have applied it to protein energetics follows.

Given a discrete variable *σ* that can take on *M* different values (*σ* = 0 … *M* − 1), any function of it can be expanded using a basis set of *M* linearly independent functions Φ = {*φ*_0_≡1, *φ*_1_, … , *φ_M_*_−1_}:

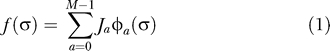
where *J_a_* are constants. A similar statement can be made about any function 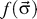 of *N* discrete variables 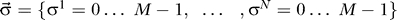 , because 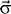 can be thought of as a discrete variable with *M^N^* possible values. Thus, to expand 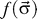 exactly, a basis set with *M^N^* functions is needed. Let vector 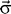 represent an amino-acid sequence with element indices of the vector corresponding to sites on the protein under study. Thus, we consider *N* sites on a protein with *M* amino acids possible at each site. Further, let function 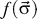 be the optimal energy of sequence 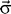 on a given backbone. According to the CE formalism [15], a particularly convenient basis set for expanding 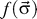 can be obtained by considering all the possible products between functions in the *N* point basis sets 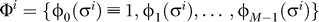 , each completely describing the sequence space at site *i*. Thus, a basis set suitable for expanding 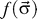 is defined in the product space of the point functions:

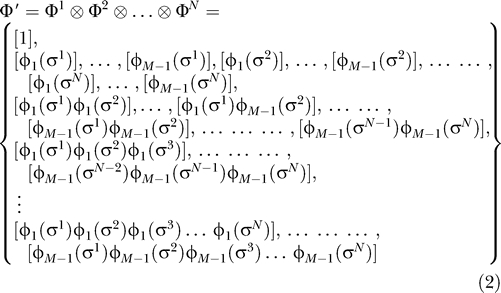
where in each row, the subscripts that index functions *φ* independently run through 1…*M* − 1 and the superscripts indexing protein sites take on all possible combinations of 1…*N*, without duplicates. Each basis function in this set (expressions in square brackets in Equation 2) depends on the amino-acid identity at either no sites (constant term), one site, two sites, and so on. We call a set of specific sites a *cluster*. Each cluster has several basis functions, or cluster functions (CFs), associated with it. For instance, any point cluster *i* (a cluster consisting of site *i*) has *M* − 1 CFs associated with it (functions *φ*_1_(*σ^i^*),…,*φ_M_*_−1_(*σ^i^*) but not *φ*_0_(*σ^i^*)≡1, which is attributed to the constant cluster). Therefore, there are a total of *N* (*M* − 1) point CFs (the second row in Equation 2) because there are *N* point clusters. Similarly, each pair cluster {*i*,*j*} has (*M* −1)^2^ CFs associated with it (*φ*_0_(*σ^i^*)*φ_k_*(*σ^j^*)≡*φ_k_*(*σ^j^*) and *φ_k_*(*σ^i^*)*φ*_0_(*σ^j^*)≡*φ_k_*(*σ^i^*) are associated with point clusters *i* and *j* for *k* > 0 and with the constant cluster for *k* = 0). Because there are *N* (*N* − 1)/2 pair clusters, the total number of pair CFs is (*M* − 1)^2^ (*N* − 1)/2 (the third row in Equation 2). For a size-*k* cluster, there are 
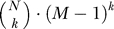
 CFs. Therefore, the total number of CFs is 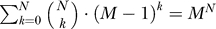 , and there are as many linearly independent CFs in the basis set as there are possible values of the discrete variable 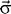 . Given the constructed basis set, we can exactly expand the energy of a sequence on the modeled backbone as:

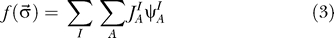
where *I* is a cluster of sites, 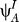 is the *A*-th CF associated with cluster *I*, and the coefficients 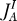 are referred to as effective cluster interactions (ECIs).

### Interpretation of the Expansion

Because the point basis set at a single AA site 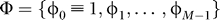 can be any set of linearly independent functions, we choose for simplicity 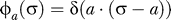 . In other words 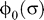 is always one, and for *a* > 0, 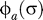 is always zero unless it is applied to the amino acid with index *a*. For any particular sequence 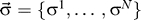 , the only CFs that remain in the expansion are of the form 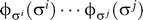 where *σ^i^*…*σ^j^* ≠ 0 (see Equation 2) and thus 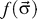 is expressed as:


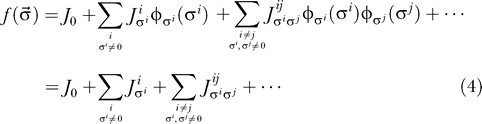


The first term in the expansion is constant and *J*_0_ can be thought of as the energy of a reference sequence. Indeed, for a hypothetical sequence 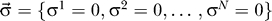 , the only surviving part of the expansion is the constant term. The amino acid that is assigned index zero at each site defines the reference sequence; for simplicity, we take this to be alanine. The ECI corresponding to higher-order terms in the expansion then define additional contributions to the energy of a sequence relative to poly-alanine. For example, 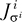 corresponds to the point contribution of amino acid *σ^i^* at site *i* relative to alanine at that site. This is the sequence context–invariant portion of an alanine-mutation energy. If there were no interactions among amino acids, point contributions and Ala-mutation energies would be equivalent. The context-dependent effects are captured by higher-order terms. For example, when interactions are present, the ECI corresponding to the terms 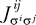 capture the effective interaction between amino acids *σ^i^* at site *i* and *σ^j^* at site *j* relative to an Ala-Ala pair. Notice, however, that for amino-acid pairs Ala-X at sites *i–j,* where X denotes any amino acid, there is no corresponding term 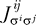 in the expansion (see Equations 3 and 4). The contribution of this interaction is captured in the point energy for amino acid X at site *i*. Therefore, the ECI corresponding to 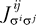 represents the pure effective interaction between the two amino acids, devoid of self contributions. This is conceptually identical to a double-mutant coupling energy—a measure well known to biochemists [23–25]. Coupling energies measure the change in stability brought about by a double mutation, corrected by the change in stability due to each of the two single mutations. If the reference sequence in our CE is poly-alanine, pair ECI correspond to double-alanine mutant coupling energies.

Even though the physics determining the conformational energy of a protein in solution is frequently modeled with only single-atom energies and pairwise atomic interactions, higher-order contributions may arise if one integrates out some degrees of freedom. For example, when modeling molecular solvation, if individual solvent molecules are replaced with a continuum high-dielectric medium, higher-order interactions are necessary to accurately describe electrostatics as a function of conformational changes in the solute [26]. Similarly, integrating out side-chain degrees of freedom and expressing energy as a function of sequence can lead to higher-order interactions between sequence variables, even though on the atomic level no more than pairwise interactions are present.

As shown in Equations 3 and 4, the CE formalism allows for arbitrarily high-order interactions (up to N-tuples) of residues. If all of the *M^N^* terms have to be accounted for, such an expansion is not very useful. However, intuition dictates that for physical systems, higher-order interactions should be less important and, thus, that ignoring them may be appropriate. If the expansion is truncated, the remaining coefficients 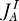 can be fit to minimize the error between the correct value of some desired fitness function and its CE approximation. Given a set of training sequences 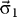 to 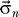 with known energies 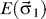 to 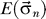 , Equation 3 defines a system of linear equations with 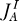 as the unknowns (each equation corresponding to one sequence).

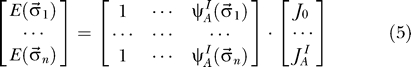
If there are more sequences than CFs, the linear system in Equation 5 becomes over-determined, and it is possible to use least-squares fitting to find the optimal values of 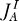 .

## Results

In principle, the method of CE can be applied to any property of a protein sequence that can be computed or measured experimentally for a large set of training examples. In this work, we expanded the energy of a sequence adopting a particular backbone conformation, which is a necessary component for protein design and some methods for fold recognition. We computed this energy in two different ways. First, using a side-chain repacking scheme and a molecular mechanics potential (giving 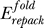 ) and second, subjecting every repacked structure to a short, continuous side-chain relaxation procedure and then re-evaluating it with a more accurate energy function that included a non-pairwise decomposable electrostatics treatment (giving 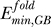 )—see Materials and Methods.

In Results we describe the application of CE to model the energetics of three different protein folds—the parallel dimeric coiled coil (an extended periodic structure), the zinc finger, and the WW domain (both aperiodic). These three structures, though small, are each of significant biological importance and have been the subject of previous protein design efforts using a variety of techniques [27–31].

### Coiled Coil

**Figure 2:**
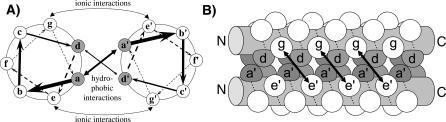
Schematic of a Parallel Dimeric Coiled Coil (A) Helical-wheel representation shows an end-on view of the structure. Opposing **a** and **d** residues interact in the core while opposing **e** and **g** residues frequently participate in electrostatic interactions. (B) Cartoon representation of the coiled coil, viewed from the side. Residues are represented as spheres. An **e** position is better located for interaction with the **g** position of the previous heptad on the opposing strand than with the **g** position of the next heptad (bold arrows). This interaction is denoted **g-e′+**, and coupling energies for it have been determined experimentally [24].

The method of CE is particularly well suited for systems dominated by local interactions, because this limits the number of clusters that need to be included. CE also has an additional benefit in periodic systems, in which modeling the energetics of a repeating unit cell can capture the behavior of the entire system. Both conditions are usually true in alloy theory, where the method is used extensively. Although proteins are rarely periodic, there are instances in which they are approximately so. An example of such a system is the α-helical coiled coil. The coiled coil is a common structural motif estimated to be present in approximately 5% of all proteins [32]. It consists of two to five right-handed helices that wrap around each other in a left-handed manner to form a super helix [33,34]. Because of this super-coiling, the backbone geometry is repeated every seven residues—a unit that is referred to as a heptad, with its residues labeled **abcdefg**. Coiled coils can either be parallel (all N termini at one end), anti-parallel (N and C termini at opposite ends), or mixed (in higher-order oligomers). In a parallel dimeric coiled coil (see Figure 2), positions **a** and **d** are located in the core of the dimerization interface, whereas positions **e** and **g** are largely solvent exposed and can form salt bridges between strands of the coiled coil. Positions **b**, **c**, and **f** are solvent exposed on the side of the helix opposite to the dimerization interface.

**Figure 3:**
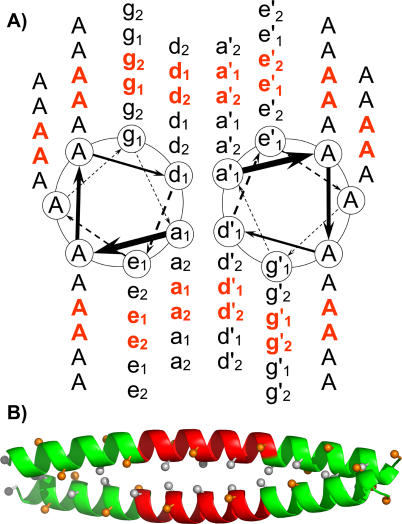
The Unit Cell Used for Modeling Coiled-Coil Interactions The entire structure consists of three copies of the sequence of the central unit cell, which is **a_1_**AA**d_1_e_1_**A**g_1_a_2_**AA**d_2_e_2_**A**g_2_** on the one strand and **a^′^_1_**AA**d^′^_1_e^′^_1_**A**g^′^_1_a^′^_2_**AA**d^′^_2_e^′^_2_**A**g^′^_2_** on the other, marked in red (A = alanine). Only positions **a**, **d**, **e**, and **g** were allowed to vary. The energy of the central unit cell was calculated as the sum of its internal interactions and half of its interactions with the bounding structure. (A) Helical-wheel diagram corresponding to the entire structure modeled, with sites in the central unit cell colored red. (B) Ribbon diagram representation of the modeled system viewed as in Figure 2B with the central unit cell colored red. Grey and orange balls represent locations of side-chain C_β_ atoms of **a**/**d** and **e**/**g** sites, respectively.

The parallel dimeric coiled coil is an extended structure, so it is reasonable to expect that only local clusters will contribute significantly to the energy expansion. Additionally, it is a periodic structure, so by accurately modeling the interactions of one structural subunit (unit cell), we can describe a coiled coil of arbitrary length. The unit cell must contain within it all interactions likely to be important. We postulated that interactions between amino acids more than one heptad apart are not significant. Thus, we modeled the unit cell as the central two-heptad section of a six-heptad dimeric coiled coil in which the flanking sequences were copies of the unit-cell sequence (see Figure 3). Because it is generally assumed that positions **b**, **c**, and **f** play only a minor role in determining the dimerization properties of coiled coils, we set these to alanine in our model. Positions **a**, **d**, **e**, and **g** were allowed to be one of 19 amino acids (all natural ones except proline, which is rare in coiled coils).

**Figure 4:**
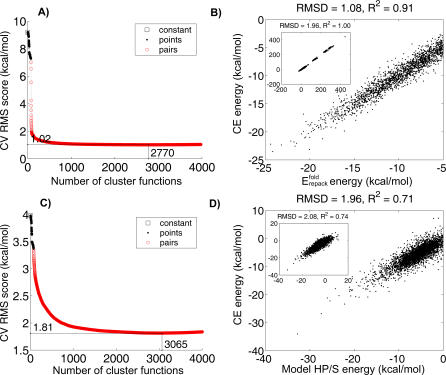
Cluster Expansion of Coiled-Coil Energies (A) and (B) refer to the CE of 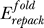 ; in (C) and (D), energies from model HP/S [35] were used. (A) and (C) represent the evolution of the CV score (the progress of the fit) as the number of CFs was increased, with the type of CF added at each point (i.e., constant, point, or pair) indicated by color. The ordering of the points is described in Materials and Methods. The set of CFs and ECI used in the final expansion was taken from the point with the minimal CV score, which is indicated on the graphs. (B) and (D) show the performance of the respective CEs on predicting energies of 4,000 random sequences not included in the training sets. Insets show the entire range of energies, whereas only sequences with reasonably low energies are shown in the main plots.

We expressed the folding energy of a parallel dimeric coiled coil (i.e., the difference between the dimer state and the unfolded monomers state) as a function of its sequence. In order to be tractable, the expansion in Equation 3 must be truncated. Consistent with our unit-cell approximation, we included only clusters involving sites no more than seven residues apart in the expansion. Further, as a starting point, we included only up to pair clusters, resulting in a total of 137 clusters. Taking into account coiled-coil symmetry (ECI for symmetry-equivalent clusters are identical [15,16]), this was reduced to one constant, four point, and 36 pair clusters with unique ECI. To find appropriate values for coefficients 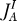 , we considered approximately 30,000 randomly generated sequences (i.e., approximately 2.5 times as many sequences as 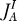 parameters being fit) and computationally predicted their structures under the assumption of a constant ideal backbone and discretized side-chain conformations [35]. This involved searching a conformational space of 10^53^ structures for an average sequence. Given optimized structures, we calculated 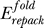 for each and used these as a training set to find optimal values for 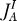 (see Materials and Methods and Figure 1). Figure 4A shows the progress of the fit accuracy, measured by cross-validation, as a function of the number and type of CFs included in the expansion. The largest drop in error, per CF, is due to point CFs. This is intuitive and consistent with the fact that there are strong amino-acid preferences at different coiled-coil heptad positions [36,37]. A few important pair CFs further reduce the error significantly, and many less-important pairs drive the error down slowly.

Figure 4B shows the performance of the resulting CE on predicting coiled-coil energies for a test set of 4,000 sequences not present in the training set. When deriving the expansion, we considered only the energy of a two-heptad unit cell, so training-set sequences were periodic with a two-heptad sequence repeated three times (see Figure 3 and Materials and Methods). The test set, however, contained non-periodic six-heptad sequences and allowed us to evaluate not only the accuracy of the CE, but also the validity of the unit-cell approximation. The overall root mean square deviation (RMSD) is 1.96 kcal/mol, whereas that for more relevant sequences (those with calculated energies below −5 kcal/mol) is 1.08 kcal/mol. This is a very small error and is in fact comparable to or better than the accuracy of the underlying energy function. Thus, for a six-heptad coiled coil, the CE formalism reduces a sequence-structure space of 10^115^ possibilities to a search of 10^61^ sequences with minimal cost in accuracy. The reduction of search space grows exponentially with the length of the coiled coil modeled.

One of the strengths of the CE approach is that, in principle, any energy function can be expanded as a function of sequence. In a previous study we found that more reasonable coiled-coil energies were obtained by allowing the structures resulting from discrete side-chain repacking to relax via several steps of continuous side-chain minimization [35]. In addition, we derived a specific physics-based energy model (HP/S) that performed well in predicting coiled-coil dimerization preferences [35]. Unlike the original energy function used above, HP/S is not pairwise decomposable at the atomic level, due to its more accurate treatment of electrostatics. We fit a CE for the HP/S energy using the same training-set sequences as before. Figure 4C shows the progress of the fit as a function of the number and type of included CFs. Again, constant, point, and pair clusters are sufficient for reasonable accuracy. Figure 4D shows the performance of the resulting CE on a set of 4,000 test sequences not included in the training set. The error for relevant sequences (those with energies below 0 kcal/mol) is 1.96 kcal/mol. Note that these energies are not strictly on an experimental scale. Our previous work has determined that stable coiled coils of five to six heptads have energies varying over 15 kcal/mol using this energy function [35], and random sequences span a range of over 40 kcal/mol; this is surely larger than the range of experimental free energies of folding.

**Figure 5:**
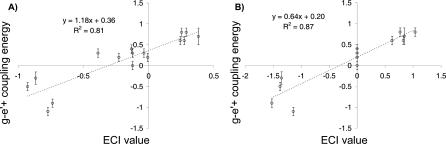
Agreement between Experimentally Measured Double-Alanine Coupling Energies and Corresponding Pair ECI The coupling energies for residues E, Q, R, and K at **g-e′+** [24] and corresponding pair ECI from the CE (in kcal/mol) are shown. (A) Energies from repacking calculations, 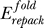 , were used to fit the CE. (B) 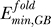 energies were used to fit CE.

Given the accuracy and simplicity of the CE functional form, the task of evaluating the energy of a sequence is reduced to several interaction table lookups, providing a significant computational advantage. However, the CE formalism is also convenient because the functional form implies that individual ECI have clear physical interpretations. Specifically, pair ECI correspond to double mutant coupling energies. Figure 5A shows the agreement between experimentally measured **g-e′+** coupling energies [24,38] (the prime designates the opposite strand and the plus sign [+] indicates the next heptad) and the corresponding pair ECI from the CE of 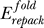 . The excellent agreement illustrates the physical interpretability of the CE. Figure 5B shows the same correspondence, but for pair ECI from the cluster expansion of HP/S energies. Because in the calculation of coupling energies the effect of the unfolded state cancels exactly, we observe a largely quantitative agreement between theory and experiment, unlike in the case with folding free energies.

### Zinc Finger

**Figure 6:**
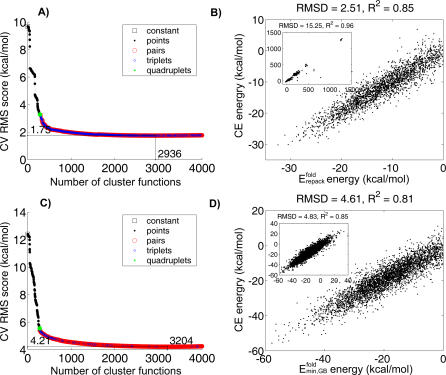
Cluster Expansion of Zinc-Finger Energies (A) and (B) refer to the CE of 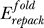 ; in (C) and (D), 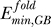 was used. (A) and (C) represent the evolution of the CV score (the progress of the fit) as the number of CFs was increased, with the type of CF added at each point (i.e., constant, point, pair, triplet, or quadruplet) indicated by color. The ordering of the points is described in Materials and Methods. The set of CFs and ECI used in the final expansion was taken from the point with the minimal CV score, which is indicated on the graphs. (B) and (D) show the performance of the respective CEs on predicting energies of 4,000 random sequences not included in the training sets. Insets show the entire range of energies, whereas only sequences with reasonably low energies are shown in the main plots.

A cluster expansion including only up to residue–pair interactions works well for the coiled coil, an extended fold in which only local interactions are likely to be important. To test whether this is a unique property of the coiled coil and whether higher-order interactions are important in more globular folds, we examined the zinc-finger motif. Zinc fingers are found in a diverse set of proteins that require coordination of one or more zinc ions to stabilize their structure [39]. Cys_2_His_2_ zinc fingers coordinate a zinc ion with two cystine and two histidine residues and are found in many DNA-binding proteins. Among these, the murine zinc finger Zif268 has been extensively studied [40]. To derive a CE describing the Zif268 fold, we defined the backbone using coordinates from the Protein Data Bank (PDB) entry 1ZAA, residues 33–60. The amino acids allowed at each site were based on the classic design by Dahiyat et al. [29] and were such that one core site was chosen from seven aliphatic amino acids, 18 surface sites varied among ten amino acids and seven interface sites were selected from 16 amino acids (a sequence space of 10^27^). This restriction gives design sequences with better physical properties while retaining a large and diverse protein design search space. Side-chain repacking was used to calculate folding energies 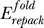 for approximately 60,000 random training sequences and a CE was derived. Results for the zinc finger are summarized in Figure 6. The progress of fitting 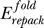 is shown in Figure 6A, where the order in which triplet and pair CFs were added is defined in Figure 1 (see Materials and Methods). In this case, triplet CFs are necessary to attain good accuracy, and it is not strictly true that pair terms contribute more significantly than triplet terms. Additionally, the contribution of point terms is relatively larger than for the coiled coil, indicating that an amino acid's contribution to the overall energy is affected significantly by the three-dimensional template of the molecule. Figure 6B shows the accuracy of the derived CE when tested on a set of 4,000 random sequences not included in the training set. The RMSD of 15.3 kcal/mol over the entire range of energies is quite high, but this is due to the large spread in energies (over 1,000 kcal/mol) caused by many of the sequences producing van der Waals clashes. As a percentage of the range, the error is quite low (<1.5%), and for the more realistic zinc-finger sequences (those with negative energies), the error is only 2.5 kcal/mol. In this case, CE reduces a sequence-structure space of 10^60^ to 10^27^ sequences.

**Figure 7:**
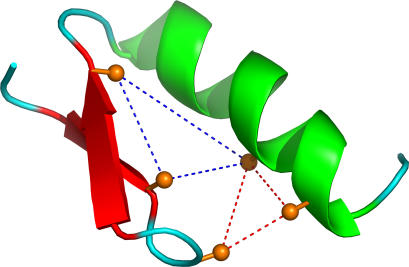
Important Triplet Clusters for the Expansion of Zinc-Finger Energies Orange balls represent the location of the C_β_ atoms of side chains. Two clusters are shown, one in red and one in blue.

To expand a more physically meaningful energy, we used approximately 30,000 structures to calculate 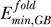 for each and used these for training. The progress of the resulting CE fit is shown in Figure 6C. Once again, triplet terms are important for attaining good accuracy. Most of the triplet CFs arise from the two triplet clusters shown in Figure 7. These are structurally compact, with CFs of significant magnitude mostly corresponding to large amino acids (such as Y, F, and W). Such clusters represent close-range interactions of bulky residues. Figure 6D shows the performance of the CE on a test set of 4,000 sequences not included in the training set. Though the agreement is still very good (*R^2^* = 0.85), the error is larger than in other cases (4.61 kcal/mol for sequences with energies ranging between 0 and −60 kcal/mol) indicating that the more complicated geometry of the domain may make the energy a more complex function of sequence.

### WW Domain

The WW domain is a protein–protein interaction motif composed of 35–40 residues. It forms the smallest known independently stable triple-stranded antiparallel β-sheet. WW domains bind proline-rich or proline-containing ligands [41]. A defining feature of this motif, from which its name is derived, is the presence of two tryptophans spaced 20–22 residues apart. Under the assumption that the statistical information encoded in multiple sequence alignments of WW domains reflects evolutionary constraints, Ranganathan and co-workers have used these statistics to engineer artificial WW domains with specific binding properties [28,42]. Protein design methods using energy functions similar to those we employ here have also been applied to this domain [31].

We derived a cluster expansion for the WW domain that captures relationships between sites that are important for folding energetics. We used the structure of human PIN1 WW domain to define backbone coordinates and chose an alphabet of amino acids at each site using a multiple-sequence alignment of WW domains from the SMART database [43]. The choices at each position covered at least 90% of all naturally occurring residues. Thus the search space is very diverse, while at the same time it excludes sequences that are grossly incompatible with the WW domain fold and not worth searching. The resulting problem had an average of 7.5 amino acids per position and a total of 1.1 × 10^27^ possible sequences. We explicitly computed structures for approximately 42,700 random sequences and estimated their folding energies.

**Figure 8:**
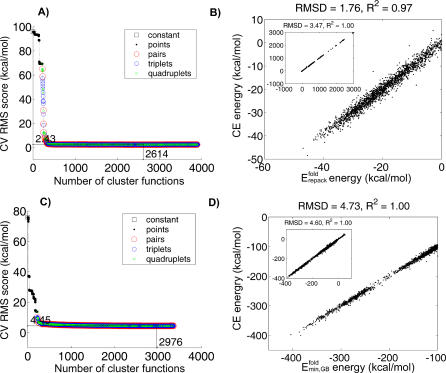
Cluster Expansion of WW-Domain Energies (A) and (B) refer to the CE of 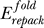 ; in (C) and (D), 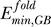 was used. (A) and (C) represent the evolution of the CV score (the progress of the fit) as the number of CFs was increased, with the type of CF added at each point (i.e., constant, point, pair, triplet, or quadruplet) indicated by color. The ordering of the points is described in Materials and Methods. The set of CFs and ECI used in the final expansion was taken from the point with the minimal CV score, which is indicated on the graphs. (B) and (D) show the performance of the respective CEs on predicting energies of 4,000 random sequences not included in the training sets. Insets show the entire range of energies, whereas only sequences with reasonably low energies are shown in the main plots.

Results of applying CE to the WW domain are summarized in Figure 8. Figure 8A shows the progress of expanding 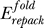 as a function of the number and type of CFs in the expansion. Similar to the zinc finger, we found that higher-order terms (11 triplet clusters and one quadruplet cluster) were necessary for good agreement. Figure 8B shows the performance on a set of 4,000 test sequences not included in the training set. The error of only 1.76 kcal/mol over a range of approximately 40 kcal/mol is impressively low and the correlation is good. Here CE reduces a sequence-structure space of 2.6 × 10^65^ to 1.1 × 10^27^ sequences.

**Figure 9:**
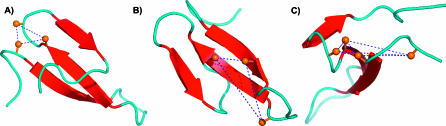
Important Higher-Order Clusters for the Expansion of WW-Domain Energies Orange balls represent the location of the C_β_ atoms of side chains. (A) A structurally compact cluster corresponding to short-range interactions. (B) A more disperse cluster arising from long-range electrostatic interactions. (C) Quadruplet cluster with many contributing CFs corresponding to a wide range of amino-acid types.

Figure 8C shows the progress of expanding 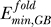 for the WW domain. Once again, higher-order interactions contribute significantly to the expansion. However, the relative contribution of point terms as compared to the case in which no minimization was done (Figure 8A) is much larger. This is likely due to the fact that many high-energy side chain–to–side chain interactions were relieved upon minimization. Several triplet clusters contribute many CFs of considerable magnitude. However, unlike for the zinc finger, for the WW domain there are two types of triplet clusters. One consists of structurally compact sites, and CFs arising from these clusters are mostly positive and correspond to large amino acids (see Figure 9A for an example). In the other, sites are more structurally dispersed, and combinations of residues producing significant CFs consist mostly of charged and polar amino acids (see Figure 9B). These two types of clusters roughly correspond to the two main classes of interactions we model—van der Waals (short range) and electrostatics (which can be long range). Additionally, there is one quadruplet cluster that seems to be important for overall accuracy—it is shown in Figure 9C. The set of amino acids at this cluster that give rise to large CFs is diverse, and it does not have a clear structural or energetic interpretation. The error of the fit, 4.7 kcal/mol (Figure 8D), is higher than before but, considering the energy range of over 300 kcal/mol, this is sufficiently accurate to be very useful.

### A Design Application and Speedup Analysis

Because the sequence-dependent energy function provided by CE is enormously simplified relative to the full physical model, it takes significantly less time to evaluate the energy of one sequence. This parameter is of critical importance in protein design, where very large sequence spaces need to be searched. We compared the amount of time it takes to evaluate the energy of one sequence either with the direct structural method or using CE (see Materials and Methods; all computations were run on 2.4-GHz CPU machines with 2 GB of memory, although memory was not a limiting factor). For the coiled-coil system considered above (a total of 48 variable sites), it took 360 s on average to repack, minimize, and re-evaluate one sequence. Using CE, it took 4 × 10^−5^ s to evaluate an approximation of that same energy, a speedup of 9 × 10^6^. For the zinc finger (a total of 26 variable sites), it took on average 70 s per sequence for the structural method and 7 × 10^−6^ seconds with CE—a speedup of 10^7^. And finally, for the WW domain (34 variable sites), the corresponding times were 70 s and 6 × 10^−6^ s—a speedup of 1.2 × 10^7^.

**Figure 10:**
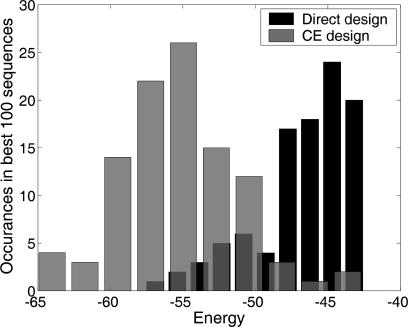
Distribution of the Energies of the Top 100 Sequences from Direct Design and CE Design The best solutions from CE design were modeled and repacked using the direct method for comparison purposes. Thus, the reported energy is that computed using the direct method for both cases. The best sequence found with CE design is significantly better than the best one from direct design. Also, the ensemble of best sequences found with CE is significantly more stable than that from direct design. This indicates that its greater speed allows CE design to reach and sample a lower-energy sequence space.

The large speed advantage of CE comes at the cost of an error in energy. In addition, deriving a CE relies on evaluating a set of training sequences with the slower, atomic-level methods and carrying out the fitting procedure. To assess the overall advantage that CE brings to protein design, we used the zinc finger as an example and carried out two design procedures. One was a sequence search driven by the “exact” energies obtained by repacking, minimizing, and evaluating every sequence (direct design). The other consisted of using the same evaluation procedure to calculate energies for a training set of random sequences, deriving a CE and performing a sequence search guided by CE energies (CE design). In an approximation of a head-to-head competition, the two methods were allowed the same amount of wall-clock time (approximately 2 d), and up to 20 processors, as follows. Direct design was allowed to sample a total of 60,000 sequences by performing 20 independent Monte Carlo runs, each with 3,000 steps (with the temperature linearly falling from 1,000 K to 298 K and the acceptance of each step governed by the Metropolis criterion [44]), which took 2 d on 20 processors. Fitting the CE required explicit modeling of approximately 30,000 sequences, which took 1 d on 20 processors. In addition, the fitting procedure (run in serial) took approximately a day of mostly human operational time (see Materials and Methods for details of the fitting procedure). Upon completing the fit, CE design was given 12 min on one processor to run 100 Metropolis Monte Carlo searches guided by CE energy, each with 10^6^ steps and the same temperature range as above. The best sequences from each of these 100 runs were then explicitly repacked, minimized, and evaluated using the original, direct energy function. Figure 10 compares energy histograms corresponding to these sequences (with their energies evaluated with the explicit energy function) and the 100 best sequences from direct design. Clearly, due to its ability to cover a considerably larger sequence space, CE discovers significantly better sequences.

## Discussion

We successfully adapted the method of cluster expansion [15], often used in alloy theory, to express the energies of proteins in several backbone conformations directly as functions of their sequences [22]. The resulting energy functions are a tremendous simplification relative to the underlying physical model, and as such offer an enormous computational speedup compared to explicit atomic-level calculations. Despite their simplicity, these functions produce energies in close agreement with those obtained through explicit calculations. Additionally, the functional form associated with the CE formalism ensures that the individual terms of the final expression are easily interpreted physically. The fact that this approach can be used in conjunction with any theoretical or experimental energy function, regardless of its complexity, makes this a very powerful general method that is likely to prove useful for many computational structural approaches.

We successfully applied CE to three model systems and illustrated its potential for computational protein design. Figure 4 shows the results for the parallel dimeric coiled coil. We found that including only up to pair interactions in the CE was sufficient for excellent agreement, giving an error of just 1–2 kcal/mol. Interestingly, several methods of scoring coiled-coil dimerization have assumed that pair interactions in sequence space are sufficient to describe the fold [36,45,46]. Additionally, many experimental studies of coiled-coil interactions have made the assumption that a pair of amino acids at a pair of sites has a roughly constant contribution, regardless of the remaining sequence environment [38,47]. The finding that a CE with only up to pair terms is sufficient to accurately describe the energy of the entire structure supports these assumptions.

One of the strengths of the CE approach is the transparency of the functional form and the consequent interpretability of the fitting coefficients. Supporting this, we demonstrate good agreement between experimentally measured coiled-coil **g-e′+** coupling energies [24] and the corresponding pair ECI from the CE (see Figure 5). These measures are not exactly equivalent, as coupling energies are measured in a specific context, whereas ECI capture an effective interaction between two residues that is independent of surrounding sequence. Practically, however, much of the context-dependence probably cancels in corrections for single-site effects.

There is a less direct correspondence between point ECI and Ala-mutation energies, which are very sensitive to environment. Additionally, self contributions to folding are more sensitive than coupling energies to the nature of the unfolded state, and modeling the unfolded state is a challenge. However, we do find qualitative agreement between point ECI and experimentally observed positional amino-acid preferences. Leucine has the most favorable point ECI at **d** positions according to the CE derived from minimized structures. Analysis of parallel dimeric coiled-coil sequences shows that Leu is by far the most common residue at position **d** [36,37,45]. Moitra et al. have further shown that in at least two slightly different sequence backgrounds, Leu is the most stabilizing aliphatic amino acid at the **d** position [48]. Based on these results, it is reasonable to propose that the observed preference for Leu at **d** positions in parallel dimeric coiled coils comes from a favorable single-body energetic contribution, as captured in the CE. Sequence analysis also suggests that Leu is the most common amino acid at the **a** position [36,45]. Accordingly, Leu has the second best point ECI at **a** according to the CE. In fact, six of the top seven most favorable amino acids based on point ECI are also among the top seven most frequently observed amino acids at **a** positions [36].

We also applied the CE approach to two more compact folds—the zinc finger and the WW domain, and these differ from the coiled coil in several respects. First, higher-order CFs are necessary for a good fit. Important triplet clusters can be either structurally compact or disperse. In compact triplets, the largest ECI correspond to combinations of large hydrophobic amino acids engaged in short-range van der Waals interactions. Examples of such clusters are shown in Figures 7 and 9A. Disperse clusters arise from long-range electrostatic interactions, and most significant ECI arise from triplets of charged and polar amino acids (see Figure 9B).

Another difference between the coiled coil and the two more globular systems is that the accuracy of the fit is better for the coiled coil. CE can attain an arbitrary degree of accuracy provided enough terms are included. However, to derive statistically meaningful ECI for high-order interactions, enough sequences are needed to provide several instances of that interaction. Thus, it was easier to derive a good fit for the coiled coil, where only up to pair clusters were required, than to identify and fit the triplet and quadruplet terms necessary to describe the zinc finger and the WW domain folds. Ultimately, the desired target accuracy is dictated by the application. For protein design, in which the goal is to find one or several good sequences, the magnitude of the error in all three systems is amply compensated by a sizeable increase in the accessible sequence space, especially given that the underlying full-detail physical models are only approximations themselves and do contain significant errors. For other applications, higher accuracy may be obtained by including more CFs and training on larger datasets, and/or by iteratively improving the CE fit by generating biased training datasets enriched with poorly fit sequences. Theoretically, because the complete expansion is exact, any desired level of accuracy can be attained. However, the cost of this (i.e., in time and memory requirements) depends on the specifics of the system under study, which is already apparent from the examples considered here. Alternatively, in cases in which the accuracy of the expansion is not high enough for direct application, CE can be used as a highly efficient filter followed by evaluation with a higher resolution energy function.

A trend seen in all three systems is that the accuracy of the CE fit is worse after minimizing the structures and evaluating them with a non-pairwise decomposable energy function. This indicates that the energy resulting from this procedure is a more complicated function of sequence. Interestingly, in these cases fewer important higher-order interactions are detected. This might indicate that structure relaxation reduces the importance of each high-order interaction, so they are harder to detect, but there could be more of them. Even though the error is larger for cases with minimization, the actual energies are more informative because they are devoid of the unphysical van der Waals clashes that often result from optimization in discrete side-chain space. In addition, the computational speedup is especially significant here, as minimization and re-evaluation are computationally expensive.

### Conclusions

The advantages offered by the CE methodology should make it widely useful in computational structural biology. We have demonstrated the application of CE to protein design problems in sequence spaces up to 10^27^. Application to fold-recognition problems of similar size should be straightforward, although the best energy function to expand may differ from that used here. In both design and fold-recognition, CE can be applied to help relieve the fixed backbone approximation by expanding energies for several variants of the same structure. Once expansions are complete, evaluation of a sequence, or of all sequences in a proteome, on each of the backbones is extremely fast. Additionally, given the interpretability of CE, cluster expansions of many closely related structures may reveal key structure determinants.

The prospect that CE may be able to provide a general tool for approaching problems in protein structure prediction and design, beyond the initial demonstrations that we present here, is exciting. Where the limits of the approach lie remains to be explored. We have shown that the type of expansion required will be sensitive to the protein fold studied and to the nature of the energy function being expanded. Large proteins will require more parameters and possibly more memory-efficient fitting procedures. It is easy to imagine many promising heuristics for choosing which parameters to fit strategically, however, and/or for partitioning larger problems into smaller ones. We hope that the modeling community will join us in exploring the boundaries of CE for their own problems of interest. The potential payoffs, as we have demonstrated here, are very large.

## Materials and Methods

### Repacking and minimization.

Energies for repacking were calculated in CHARMM based on parameter set 19 [49]. The energy function consisted of van der Waals energy (with atomic radii scaled to 90%), dihedral angle torsion energy, screened electrostatic interactions given by a distance-dependent dielectric model, and desolvation energy given by the EEF1 model [50,51]. We treated the unfolded state by ignoring all side chain–to–side chain interactions and treating each side chain on a five-residue stretch of its local native backbone. Rotamers were taken from the Dunbrack 2002 rotamer library [52]. We used our implementation of the dead end elimination (DEE) and A* branch and bound algorithms [53–58] to find the optimal structure for each sequence. Given this structure, we calculated its folding energy 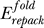 using the potential used for repacking. To compute more accurate energies (devoid of large uninterpretable steric clashes and with better electrostatics), we subjected the solutions obtained with DEE to continuous side-chain minimization in CHARMM (ten cycles of steepest-descent minimization and ten cycles of adopted-basis Newton-Raphson minimization). The resulting structures were evaluated with an alternate energy function in which 100% radii were used for van der Waals calculations, and screening of electrostatic interactions was modeled using the Generalized Born model with “perfect” Born radii [59] computed using the program PEP [60] (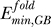 ). For the zinc finger and WW domain, the same penta-peptide representation of the unfolded state as before was used for calculating reference energies. For the coiled-coil system, additional modifications were made to the unfolded state according to an energy model previously shown to perform well in recognizing coiled-coil dimerization preferences (model HP/S) [35].

### The coiled-coil unit cell.

To derive a scoring function for coiled coils of arbitrary length, we expanded the energetics of a repeating structural element (unit cell). We postulated that interactions between amino acids more than one heptad apart in a coiled coil would not be appreciable and so did not include clusters corresponding to these interactions in the CE. The unit cell was chosen to be a two-heptad dimeric parallel coiled coil (see Figure 3). Additionally, to avoid edge effects, we used a periodic boundary condition for the backbone structure and sequence (see Figure 3). Each periodic six-heptad training-set sequence was repacked as specified above. CE was fit to just the energy of the central unit cell (all of the unit cell self energy and half of all interactions between the unit cell and the rest of the molecule), which allowed each interaction type to be counted exactly once. Thus the resulting ECI map exactly onto the energies of the corresponding interactions and can be applied to non-periodic sequences.

### Cluster expansion fitting.

If energies for enough sequences are available, 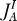 can be solved for by standard fitting procedures (see Equation 5). We used the method of pseudo-inverse [61] to perform least-squares fitting with an exponential weighting reducing the contributions of the less meaningful high-energy sequences. Therefore, for *n* CFs, the fitting procedure has a worst-case asymptotic running time of *O*(*n* ^3^) and memory requirement of *O*(*n* ^2^). Determining which of the *M^N^* CF terms to keep in the fitting is not trivial (*M* is the number of residues possible at each site and *N* is the number of sites; for simplicity, we assume all sites to have the same number of possibilities). Although one may be guided by the notion that point terms are more important than pairs, which in turn are more important than triplets and so on, this is not always true. We address the problem using the cross-validation (CV) score rather than the RMSD to guide the fitting procedure. The CV score is the average error with which each sequence is predicted when left out of the fitting, and is a good measure of predictive power. When more CFs are included, the RMSD score decreases, whereas the CV score might increase (i.e., possible over-fitting) if the CFs are not physically relevant.

The fitting procedure used was as follows (see Figure 1). The number of sequences in the training set was chosen to be in the range of 1.5–2.5 times the expected number of parameters in the fit (i.e., the number of parameters required to model up to all pair interactions). The constant and point CFs were initially included in the CF pool and used to compute a baseline value of the CV score; all pair CFs were considered as candidates for inclusion into the pool. For each pair cluster {*i*,*j*}, we considered all CFs associated with it (each corresponding to the contribution of a pair of amino acids) one at a time, and only those pair CFs that decreased the CV score were added to the pool. Because the contribution of a new CF (and its effect on the CV score) in general depends on the CFs that are already present, the order in which pair CFs are considered for inclusion into the pool is important. To determine a meaningful order, we first performed a fit with all pair CFs (in addition to the constant and points) to obtain fitting parameters *J_i_* for each *CF_i_*. Pair CFs were then considered in the order of decreasing *|J_i_|*. Once all pair CFs were considered for inclusion, it was determined whether the quality of the fit (i.e., the magnitude of the CV error) was satisfactory. If it was not, we used the characteristics of poorly fit sequences Ω:| Δ*E* | >*D kcal*/*mol* (i.e., those sequences with error larger than *D* kcal/mol, where *D* was 10 for unrelaxed cases and 5–6 for relaxed ones) to locate important higher-order clusters (triplets and quadruplets). We calculated the information content *I^i^* = ln(*M*)−*S*(*p*(*σ^i^*|Ω)) for each site *i* and *I^i^* ^,*j*^= ln(*M* ^2^)−*S*(*p*(*σ^i^σ^j^*|Ω)) − *I^i^* − *I^j^* for each pair of sites {*i*,*j*} out of the amino-acid distribution in Ω. The terms *p*(*σ^i^*|Ω) and *p*(*σ^i^σ^j^*|Ω) are the amino-acid distributions at site *i* and at the pair of sites {*i*,*j*} in the sequence profile Ω, respectively, and 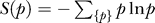 denotes the entropy of a probability distribution. Usually only a few sites had significant point information content. Triplet and/or quadruplet CFs among sites with significant pair information content were manually added to the pool. The number of training sequences was increased (i.e., energies for more sequences were explicitly calculated) if the number of fitting parameters exceeded the number of sequences. For the un-relaxed cases with the zinc finger and the WW domain, the newly considered sequences were biased to include the amino-acid pairs over-represented in poorly fit sequences. All pair CFs in addition to the selected higher-order CFs formed the new set of candidates. The procedure for considering candidate CFs one at a time was repeated as above, and a final CV score was derived.

### Zinc-finger design exercise.

The energy models employed in this study do not account for protein solubility. Additionally, the rather crude unfolded state models make it difficult to properly estimate the overall relative point contributions of different amino acids at a given site. To get around these problems, we performed fixed composition design—an optimization problem in which amino-acid composition is held constant, but the sequence is free to change under this constraint [62]. This allows one to specify a reasonable composition that ensures likely solubility while relying on the optimization process to pick a permutation particularly well suited for the given backbone. An additional advantage is the cancellation of the unfolded state energy (assuming a strict composition dependence) across different sequences.

We used the zinc-finger sequence designed by Dahiyat and Mayo [29] (QQYT AKIK RTFR NQKQ LRDF IEKF KR), which has been experimentally characterized, to fix the amino-acid composition of our design. Note that because this sequence is quite heterogeneous, the search space of all unique permutations, 8.6 × 10^20^, is very large and the design problem is still challenging. Each step of a Monte Carlo search in this fixed composition space amounted to picking two sites at random and swapping amino acids between them (if they were not already the same). Two Monte Carlo searches were run—one using repacking, minimization, and re-evaluation according to 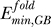 to score each sequence (direct design) and the other using CE equivalent of the same energy function (CE design). The DEE and A* branch and bound algorithms for repacking [53–58] were implemented in C. CHARMM [49] was used for continuous side-chain minimization and calculation of the van der Waals and EEF1 portions of the potential. PEP [60] was used to calculate atomic Born radii. A wrapper script that combined these steps for each sequence was written in Perl. Sequence design code was written in C to use MPI (http://www-unix.mcs.anl.gov/mpi) and was distributed over 20 CPUs. The program for searching using CE was written in C without parallelization.

## Supporting Information

### Accession Numbers

The Protein Data Bank (http://www.rcsb.org/pdb) accession number for the human PIN1 WW domain is 1PIN and for the murine zinc finger Zif268 is 1ZAA; the SMART database (http://smart.embl-heidelberg.de) accession number for the WW domain is SM00456.

## Abbreviations

CE: cluster expansion
CF: cluster function
CV: cross-validation
ECI: effective cluster interaction
PEEF: physical effective energy function
RMSD: root mean square deviation
SEEF: statistical effective energy function
